# Differentiation inducing factor 3 mediates its anti-leukemic effect through ROS-dependent DRP1-mediated mitochondrial fission and induction of caspase-independent cell death

**DOI:** 10.18632/oncotarget.8319

**Published:** 2016-03-24

**Authors:** Alix Dubois, Clemence Ginet, Nathan Furstoss, Amine Belaid, Mohamed Amine Hamouda, Wedjene El Manaa, Thomas Cluzeau, Sandrine Marchetti, Jean Ehrland Ricci, Arnaud Jacquel, Frederic Luciano, Mohsine Driowya, Rachid Benhida, Patrick Auberger, Guillaume Robert

**Affiliations:** ^1^ INSERM U1065 Centre Méditerranéen de Médecine Moléculaire, Nice, France; ^2^ Team 2: Cell Death, Differentiation, Inflammation and Cancer, Nice, France; ^3^ Equipe Labellisée Fondation ARC, Paris, France; ^4^ Université de Nice Sophia Antipolis, Nice, France; ^5^ Institut de Chimie de Nice (ICN), UMR 7272, Nice, France; ^6^ CHU de Nice, Service D'Hématologie Clinique, Nice, France; ^7^ Team 3: Regulation of Caspase Dependent and Independent Cell Death, Nice, France

**Keywords:** DIF-3, mitochondria fission, cell death, autophagy, leukemia

## Abstract

Differentiation-inducing factor (DIF) defines a group of chlorinated hexaphenones that orchestrate stalk-cell differentiation in the slime mold *Dictyostelium discoideum* (DD). DIF-1 and 3 have also been reported to have tumor inhibiting properties; however, the mechanisms that underlie the effects of these compounds remain poorly defined. Herein, we show that DIF-3 rapidly triggers Ca^2+^ release and a loss of mitochondrial membrane potential (MMP) in the absence of cytochrome c and Smac release and without caspase activation. Consistently with these findings, we also detected no evidence of apoptosis in cells treated with DIF-3 but instead found that this compound induced autophagy. In addition, DIF-3 promoted mitochondrial fission in K562 and HeLa cells, as assessed by electron and confocal microscopy analysis. Importantly, DIF-3 mediated the phosphorylation and redistribution of dynamin-related protein 1 (DRP1) from the cytoplasmic to the microsomal fraction of K562 cells. Pharmacological inhibition or siRNA silencing of DRP1 not only inhibited mitochondrial fission but also protected K562 cells from DIF-3-mediated cell death. Furthermore, DIF-3 potently inhibited the growth of imatinib-sensitive and imatinib-resistant K562 cells. It also inhibited tumor formation in athymic mice engrafted with an imatinib-resistant CML cell line. Finally, DIF-3 exhibited a clear selectivity toward CD34^+^ leukemic cells from CML patients, compared with CD34^−^ cells. In conclusion, we show that the potent anti-leukemic effect of DIF-3 is mediated through the induction of mitochondrial fission and caspase-independent cell death. Our findings may have important therapeutic implications, especially in the treatment of tumors that exhibit defects in apoptosis regulation.

## INTRODUCTION

Mitochondria are essential cellular organelles because of their crucial roles in cellular metabolism and respiration [1–3]. In addition, mitochondria also act as indispensable regulators of cell death via the release of cytochrome *c* and other proapoptotic factors that are necessary for the induction of apoptosis [4, 5]. Mitochondria are highly dynamic organelles that can change in shape and size and move to different locations within the cell, depending on both cellular circumstances and stimuli [6]. Indeed, mitochondrial morphology is adjusted and finely regulated through an exquisite balance between fusion and fission processes [7]. Importantly, unbalanced mitochondrial dynamics have been implicated in a number of human pathologies, including neurodegenerative disorders [8] and cancer [9, 10].

Mitochondrial fusion and fission processes are orchestrated through the opposite actions of the family of large GTPase dynamin proteins [11]. In mammalian cells, mitochondrial fusion is controlled by mitofusins 1 and 2 (MFN1/2) and optic atrophy 1 (OPA1), whereas fission is driven by dynamin-related protein 1 (DRP1) [12, 13]. DRP1 is recruited from the cytoplasm to the mitochondria at the sites of scission [14]. The activity of DRP1 is regulated by post-translational modifications. Phosphorylation of DRP1 at Ser^637^ by cyclic AMP-dependent protein kinase (PKA) impairs DRP1 translocation to the mitochondria [15], whereas calcineurin-dependent dephosphorylation of the same residue enhances its recruitment to the mitochondria [16]. Importantly, the putative phosphoserine/threonine phosphatase (PGAM5) in the mitochondrial outer membrane has recently been reported to play an important role in the initiation of necrosis by dephosphorylating DRP1-Ser^637^ and promoting DRP1 mitochondrial translocation [13]. In addition, phosphorylation of DRP1 at Ser^616^ by cyclin-dependent kinase-1 (CDK1) during mitosis promotes mitochondrial fission [17].

During apoptosis, mitochondria undergo important morphological alterations, transitioning from an intricate (tubular) network to punctate fragments. There is also evidence that mitochondrial fission plays an active role in apoptosis [18, 19], autophagic cell death [20, 21] and necroptosis [13]. Indeed, DRP1-induced excessive mitochondrial fission causes programmed cell death, and the inhibition of DRP1 by various means delays this process. Finally *Khasatus et al.* have recently reported that mitochondrial fission driven by DRP1 enhances tumor growth and that DRP1 may be a target of interest in treating MAP kinase-driven cancer [22]. It appears that the process of mitochondrial fission may induce cell death or contribute to cellular proliferation depending on the cell type and the intensity of the stimulus.

DIF-1 (1-(3,5-dichloro-2, 6-dihydroxy-4-methoxyphenyl) hexan-1-one) and DIF-3 (1-(3-dichloro-2, 6-dihydroxy-4-methoxyphenyl) hexan-1-one) belong to a family of morphogens required for stalk-cell differentiation in DD [23]. DIF-1 and DIF-3 exert potent anti-leukemic effects in several cancer cell lines, the latter being more potent than the former [24]. Intensive efforts have been dedicated to the characterization of the mechanisms of action of these DIFs [24–27]. Recent studies have shown that DIF-1 and DIF-3 inhibit proliferation by suppressing the Wnt/β–catenin signaling pathway via the activation of glycogen synthase kinase-3β (GSK3β). Importantly the DIF-1/3-mediated activation of GSK3β and dual-specificity tyrosine-(Y)-phosphorylation-regulated kinase 1β (DYRK1β) triggers the phosphorylation of cyclin D1 and its degradation via the proteasome pathway, an event that may partially explain the anti-proliferative effects of DIFs [28]. Nevertheless, the exact mechanism by which DIF-1/3 kills tumoral cell lines remains poorly defined. In the present study, we investigated the mechanism of action of DIF-3 *in vitro* and *in vivo*. We first established that DIF-3 induced a very rapid loss of mitochondrial membrane permeabilization (MMP) in the absence of cytochrome c and Smac release, and accordingly, DIF-3 failed to efficiently activate caspases and induce apoptosis. Consistently with these findings, we found that zVAD-fmk, a pan-caspase inhibitor did not protect cells from the deleterious effect of DIF-3. Importantly, we established that DIF-3 promoted the release of calcium into the cytoplasm and the phosphorylation of DRP1 at Ser616. DIF-3-mediated DRP1 phosphorylation resulted in its relocation from the cytoplasm to the mitochondria, an event that culminated in mitochondrial fission and cell death. Inhibition of DRP1 with small siRNA or a pharmacological inhibitor abrogated mitochondrial fission and conferred protection against DIF-3-mediated cell death. Finally, we found that DIF-3 promoted tumor regression in athymic mice transplanted with the imatinib-resistant K562 CML cell line. We conclude that DIF-3 exerts its potent anti-leukemic effect on cancer cells through DRP1-mediated mitochondrial fission and the induction of cell death.

## RESULTS

### The anti-leukemic effect of DIF-3 on CML cells is caspase-independent

**Figure 1:**
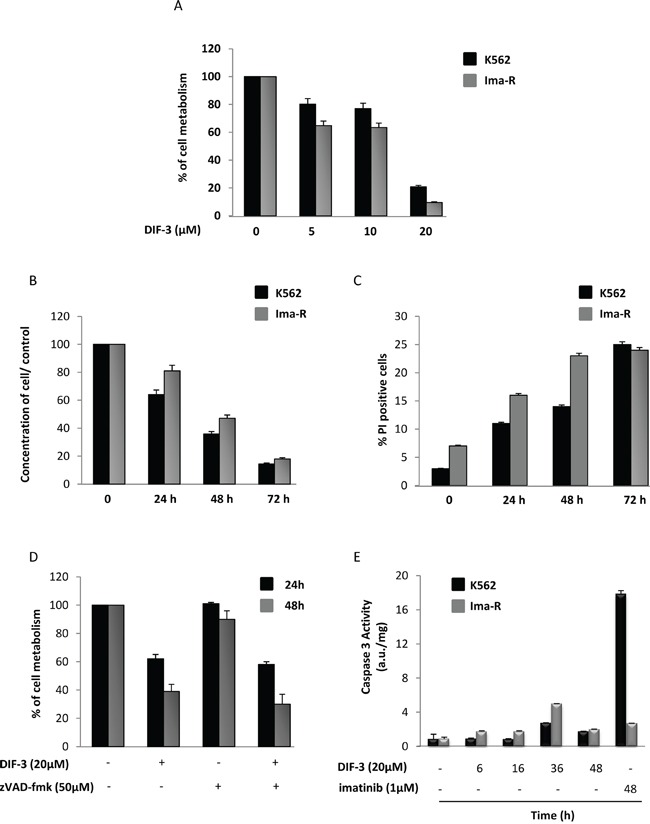
DIF-3 induces caspase-independent cell death **A.** K562 and Ima-R cells were incubated for 48 h at 37°C with increasing concentrations of DIF-3. Cellular metabolism was measured by the XTT assay, as described in the Materiel and Methods section. The results are means ± SD of 3 different measurements performed in triplicate. Error bars = 95% confidence intervals. **B.** K562 and Ima-R cells were incubated at 37°C with 20 μM DIF-3. Then, cellular concentrations were evaluated at 24, 48 or 72 h with a cytometer and compared with the initial concentrations. **C.** K562 and Ima-R cells were treated as described in Figure 1B, and cells were stained at the indicated time using propidium iodide to analyze cell death with a cytometer. **D.** K562 cells were or were not pretreated with 50 μM *Z-VAD*-fmk and incubated with 20 μM DIF-3 for 24 and 48 h. Cellular metabolism was measured by using the XTT assay, as described in the Materiel and Methods section. **E.** K562 and Ima-R cells were incubated at 37°C with 20 μM DIF-3 at the indicated time or with 1 μM imatinib (as the positive control). Cells were harvested, washed, and lysed in caspase buffer. Caspase-3 activity was evaluated in quadruplicate using Ac-DEVD-AMC as a substrate. To allow for the specific assessment of caspase activity, hydrolysis was followed over time in the presence or the absence of 10 mM Ac-DEVD-CHO. The results are expressed as arbitrary units (a.u.) per mg of proteins and are the means ± SD of 4 independent experiments performed in quadruplicate. Error bars = 95% confidence intervals.

Starting with methoxyphenol, we synthetized different DIF derivatives, including DIF-1 and DIF-3 (Supplementary Figure S1A). A dechlorinated derivative of DIF-3 and two elongated DIF-1 and DIF-3 molecules were also produced. All compounds, except 4-methoxyphenol, were shown to induce a very rapid loss of MMP (Supplementary Figure S1B) and a reduction in cellular metabolism at 24 h (Supplementary Figure S1C) at doses in the low-micromolar range in the K562 CML cell line. Due to DIF-3′s potent effect and its physiological occurrence in Dictyostelium, it was used for all the subsequent experiments. DIF-3 inhibited cellular metabolism in the CML cell line K562 with an IC50 of 11.2 μM (Figure 1A and Supplementary Figure S2A). DIF-3 was also highly efficient in its effects on a well-characterized imatinib-resistant variant of K562 cells [29–32]. The inhibition of cellular metabolism was accompanied by a net decrease in cell count that was maximal 72 h after the addition of DIF-3 (Figure 1B). This decrease in cell count reflected increased cell death, as assessed by propidium iodide staining (Figure 1C). In addition, DIF-3 efficiently killed JURL-MK1 and LAMA-84 cells, confirming the anti-leukemic potential of this molecule in two other well-characterized CML cell lines (Supplementary Figure S3).

Importantly, pretreatment of K562 cells with the pan-caspase inhibitor Z-VAD-fmk failed to affect the inhibitory effect of DIF-3 on cellular metabolism, strongly suggesting that DIF-3-mediated cell death is independent of caspase activity (Figure 1D). Accordingly, DIF-3 exerted only a marginal induction of caspase-3 activity compared with the effects of imatinib, which is the most frequently used compound to treat CML and is also known to be a potent inducer of apoptosis in K562 cells [33] (Figure 1E).

### DIF-3 inhibits the clonogenic potential of K562 cells and exhibits specificity for CD34+ from CML patients

**Figure 2:**
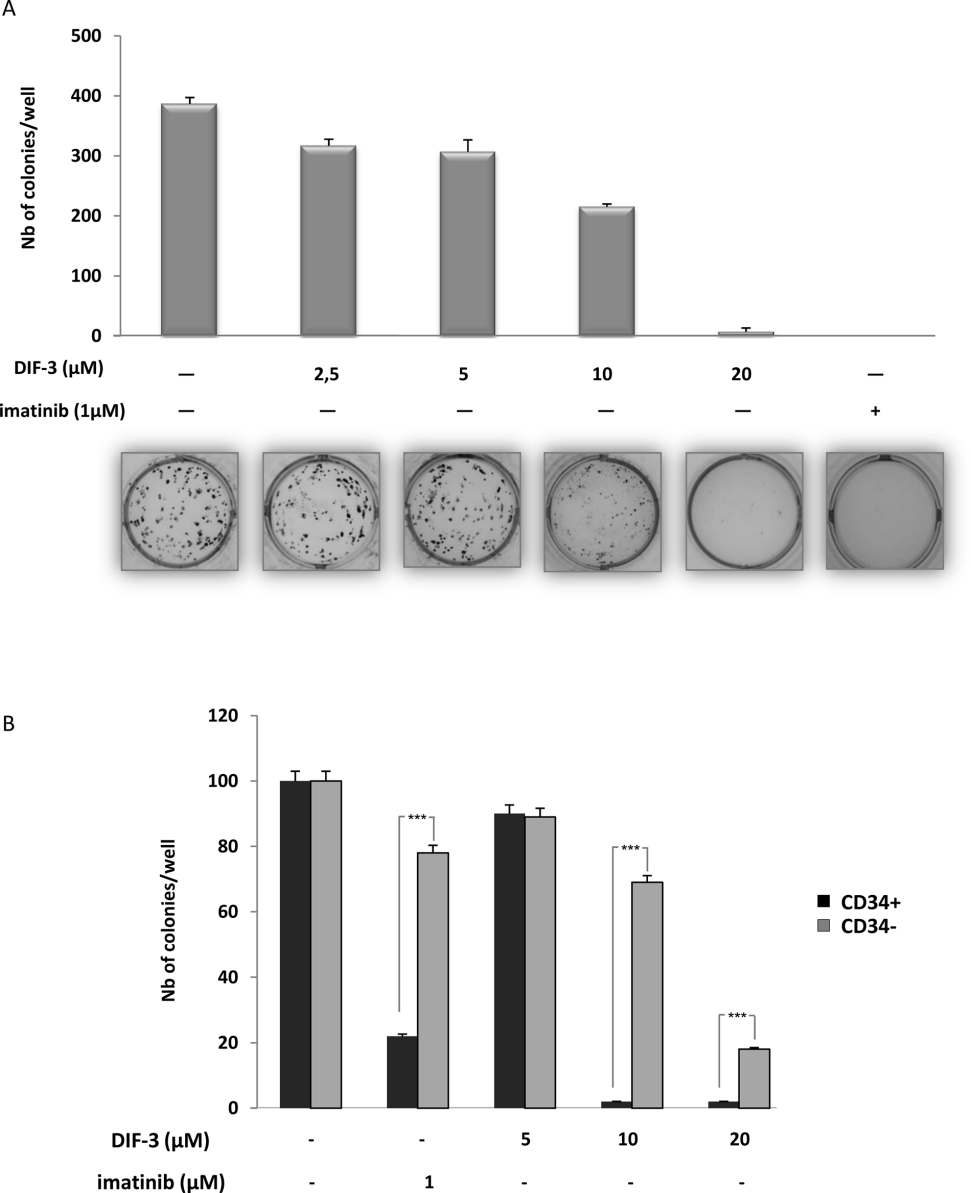
DIF-3 induces inhibition of colony formation in both K562 cell lines and primary CML cells **A.** DIF-3, ranging from 2.5-20 μM, was added to K562 CML cell lines growing in semi-solid methyl cellulose medium (0.5 × 10^3^ cells/ml). Colonies were detected after 10 days of culture by addition of 1 mg/ml of the MTT reagent and were scored using ImageJ quantification software. The results are expressed as the number of colony-forming cells per well after drug treatment. The results are the means ± SD of 3 different measurements performed in triplicate. Error bars = 95% confidence intervals. **B.** Various concentrations of DIF-3 were added to sorted CD34+ or CD34- primary cells from one CML patient collected at diagnosis. Cells (10^3^ cells/ml) were grown in a semi-solid methylcellulose medium. After ten days in culture, MTT reagent (1 mg/ml) was added to the cell culture and the number of cell colonies was determined using ImageJ quantification software.

We next investigated the effect of increasing doses of DIF-3 on K562 cell colony formation in a methylcellulose matrix, which reflects the proliferative capacity of the cell line. As shown in Figure 2A, DIF-3 induced a dose-dependent inhibition of the number of K562 cell colonies, with an IC50 of 12.5 μM (Supplementary Figure S2B). At 20 μM, DIF-3 was as effective as treatment with 1 μM imatinib, which is the leading treatment for CML patients to inhibit the clonogenic potential of K562 cells. Importantly, DIF-3 was found to be more effective on CD34+ cells from a CML patient at diagnosis than on their CD34- cell counterparts, highlighting the reason for the potential interest in this molecule as a therapeutic agent (Figure 2B).

### DIF-3 induces rapid mitochondrial fission and alterations in mitochondria morphology

**Figure 3:**
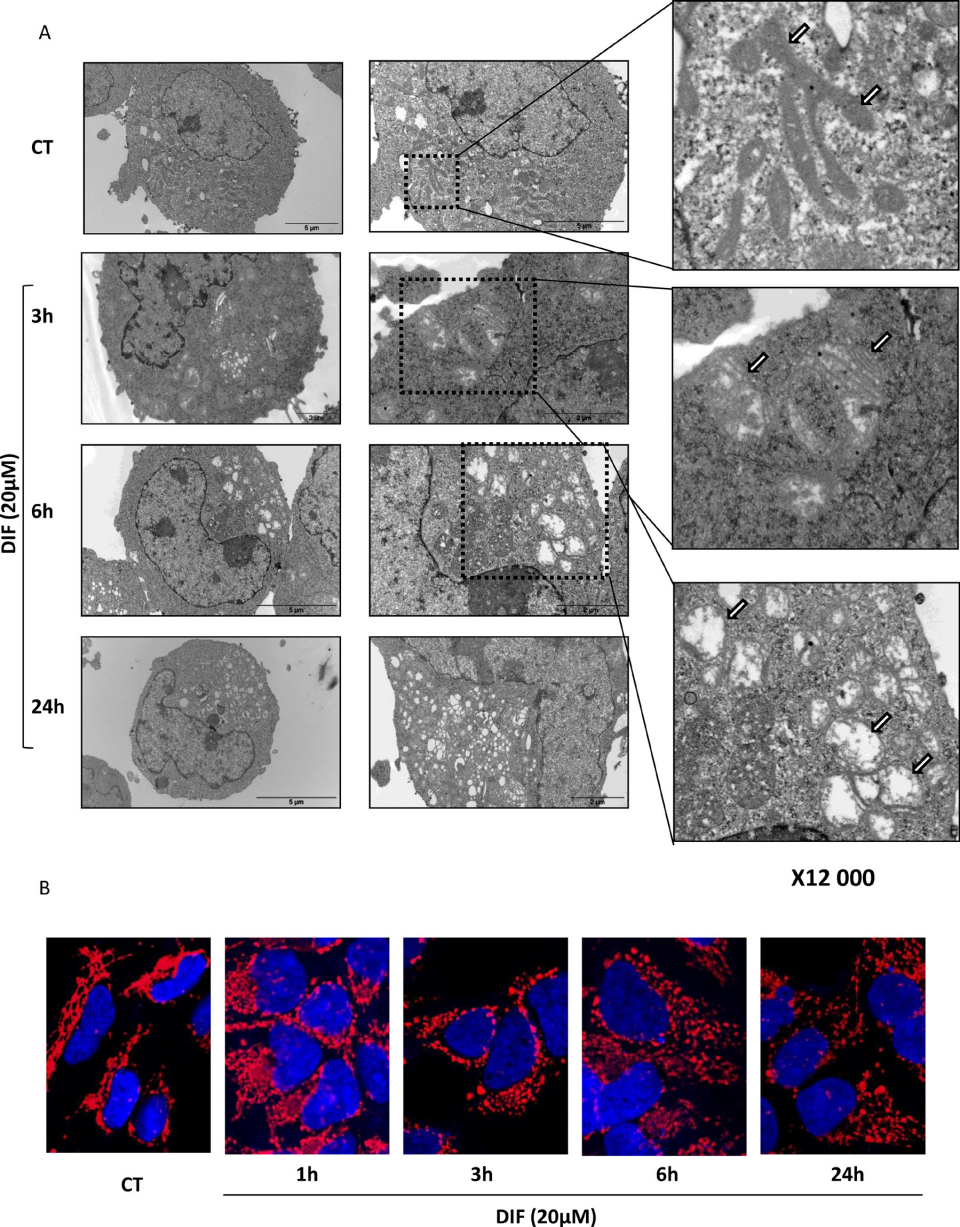
DIF-3 rapidly induces morphological changes and alterations in intramitochondrial structures **A.** Electron microscopy images showing ultrastructural features of a representative control cell and the morphological features of autophagy in K562 cells treated with 20 μM DIF-3 for 3, 6 and 24 h. Cells were observed at different magnifications (x 2 500, x 6 000 and x 12 000). M= mitochondria. **B.** HeLa cells were incubated at 37°C with 20 μM DIF-3. After 1, 3, 6, or 24 h, cells were fixed, permeabilized, and successively incubated with anti-HSP60 antibodies and a secondary antibody conjugated to red fluorochrome. The nuclei were stained with DAPI reagent (5 mg/ml). Antibody localization was visualized via confocal microscopy.

Electron microscopy images of K562 cells treated for various times with 20 μM DIF-3 are presented in Figure 3A. Untreated cells presented a characteristic network of intact and highly intricate mitochondria (Figure 3A, upper right panel). Three hours after the addition of DIF-3, mitochondria exhibited an abnormal morphology and were present as individual organelles (Figure 3A, middle right panel). At this time, there was a clear swelling of the mitochondria. At 6 h, most mitochondria were characterized by a complete absence of crests (Figure 3A, lower right panel, indicated by arrows). At 24 h after DIF-3 addition, no intact mitochondria were detectable (Figure 3A). Our data clearly show that DIF-3 interferes with mitochondrial homeostasis and suggest a role of this differentiating factor in the fusion/fission processes of mitochondria. To further investigate the role of DIF-3 in mitochondrial homeostasis, HeLa cells were treated for various times with this compound. Cells were next fixed and stained with an anti-HSP60 mAb to analyze the reorganization of mitochondria following the addition of DIF-3. In untreated cells, mitochondria were organized as a characteristic network of highly intricate (tubular) organelles, as expected from the electron microscopy images (Figure 3A). Reorganization of the mitochondrial network was detectable as soon as 1 h after the addition of DIF-3 (Figure 3B), and a clear individualization of mitochondria was observed at 3 h (Figure 3B). Finally, the swelling of individual mitochondria was clearly detectable 6 h and 24 h after the addition of DIF-3 (Figure 3B and Supplementary Figure S4). Importantly, confocal microscopy images confirmed the results obtained using electron microscopy. Collectively, our findings indicate that DIF-3 triggers mitochondrial fission, both in K562 and HeLa cells.

### DIF-3 inhibits mTOR signaling and induces autophagy in K562 CML cells

**Figure 4:**
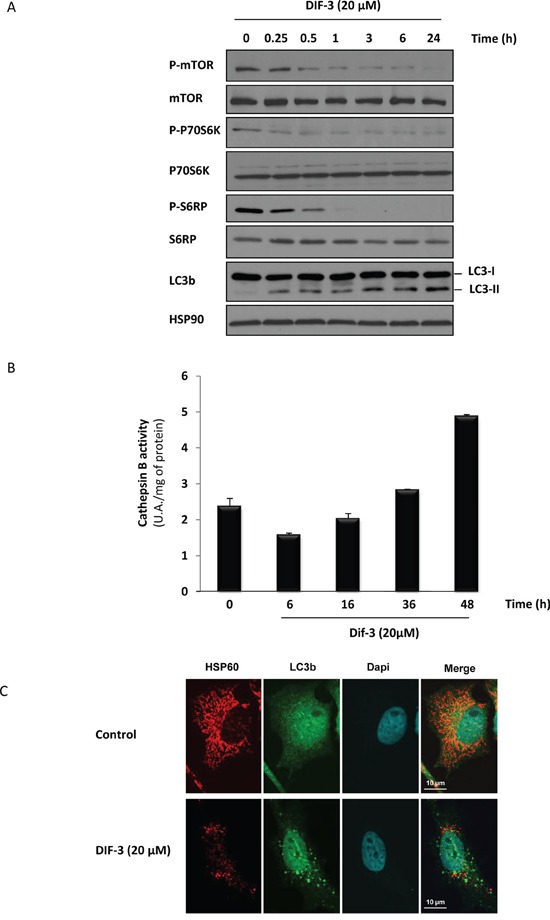
DIF-3 induces hallmarks of autophagy in K562 cells **A.** K562 cells were treated with 20 μM DIF-3 for the indicated times. Whole-cell lysates were prepared, and the expression of mTor, P70S6 kinase, S6 ribosomal protein and their phosphorylated forms, as well as LC3-b, were visualized via western blotting. HSP90 was used as a loading control. **B.** K562 cells were incubated with 20 μM DIF-3 for the indicated times at 37°C, and cathepsin B activity was evaluated in the presence or absence of CA-074Me, as described in the Materials and Methods section. The results, expressed as arbitrary units (a.u.) per mg of proteins, are the means ± SD of 4 independent experiments performed in quadruplicate. Error bars = 95% confidence intervals. **C.** HeLa cells were incubated at 37°C with 20 μM DIF-3. After 48 h, cells were fixed, permeabilized, and successively incubated with anti-HSP60 and LC3b antibodies and secondary antibodies conjugated to red or green fluorochromes, respectively. The nuclei were stained with DAPI reagent (5 mg/ml). Antibodies localization was visualized via confocal microscopy.

The results illustrated in Figure 3 show that DIF-3 alters the mitochondrial network and induces a loss of mitochondrial cristae integrity in K562 cells. To further characterize the mechanism of action of DIF-3, we next analyzed the activation of the mTOR pathway. DIF-3 induced the rapid dephosphorylation of mTOR at Ser2481. Dephosphorylation of mTOR was detected as soon as 15 min after DIF-3 addition and was maximal within 1 h. As expected, the dephosphorylation of P70S6K at Ser371 and ribosomal protein S6 at Ser235/Ser236 paralleled that of mTOR. Together, these data clearly demonstrate that DIF-3 inhibits the mTOR pathway and leads to a rapid and robust conversion of LC3-I to LC3-II (Figure 4A). Increased autophagy was accompanied by the late activation of cathepsin B, as previously described using other pro-autophagic stimuli [34] (Figure 4B).

To confirm that LC3-II accumulation was concomitant with mitochondrial disorganization, we used confocal microscopy to analyze HeLa cells that either were or were not stimulated with DIF-3 and were then stained with anti-LC3 and anti-HSP60 antibodies. As expected, we observed LC3-II conversion, as shown by the appearance of specific dots in the cytoplasm and a disruption of the mitochondrial network (Figure 4C). The effect of DIF-3 on the conversion of LC3 was not accompanied by an increase in autophagic flux, because no further accumulation of LC3-II was detected in the presence of bafilomycin A1, which did induce a strong accumulation of LC3-II (Supplementary Figure S5) on its own, thus strengthening our previous results.

In agreement with the above results, silencing LC3 or ATG5 by specific siRNA failed to counteract the anti-leukemic effect of DIF-3 on K562 cell death (Supplementary Figure S6), indicating that autophagy is not involved in this process.

### DIF-3 induces MMP, a decrease of ATP content and an induction of ROS production in K562 cells

**Figure 5:**
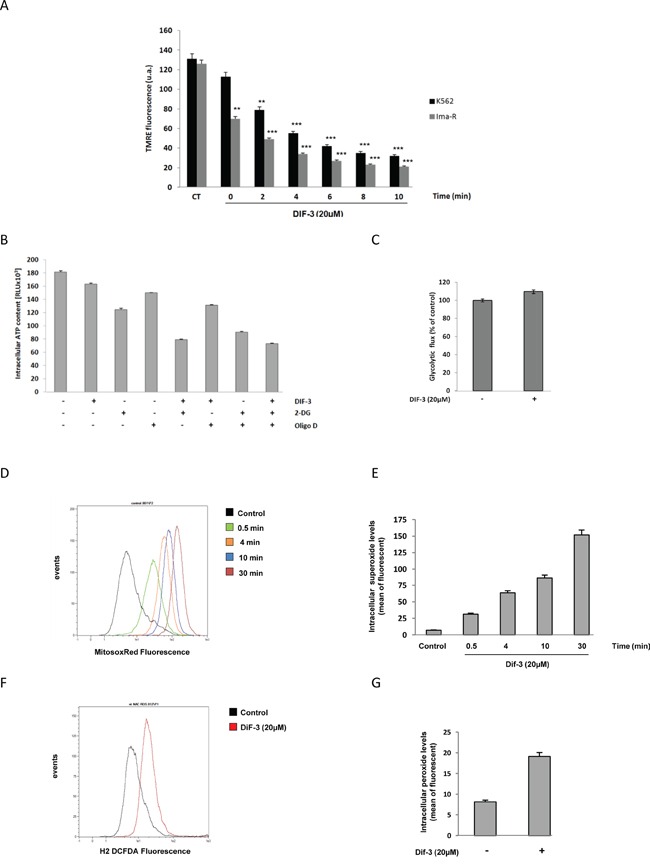
Effects of DIF-3 treatment on mitochondrial function **A.** K562 and Ima-R cells were loaded with TMRE and then stimulated with 20 μM DIF-3. Then, TMRE fluorescence was monitored with a cytometer at 2, 4, 6, 8 and 10 min. **B.** K562 cells were treated for 6 h with 20 μM DIF-3, 10 mM 2-DG, and 10 ng/ml oligomycin D. Compounds were added alone or in combination with each other. Then, intracellular ATP content was evaluated with a luciferase assay (ATPlite, Perkin Elmer). **C.** K562 cells were treated for 6 h with DIF-3 20 μM, and glycolytic rates were evaluated by quantifying of the conversion of {5-^3^H}-D-glucose to ^3^H2O, as described in the Materials and Methods section. **D.** K562 cells were loaded with MitosoxRed, stimulated with 20 μM DIF-3 and mitochondrial reactive oxygen species were quantified at 0.5, 4, 10 and 30 min post-treatment. **E.** Histograms represent the means ± SEM of mitochondrial reactive oxygen species content. **F.** K562 cells were loaded with DCF-DA and stimulated with 20 μM DIF-3 for 30 min. Cellular reactive oxygen species were quantified with a cytometer. **G.** Histograms represent the means ± SEM of cellular reactive oxygen species content.

Because DIF-3 triggered rapid alterations of the mitochondrial network in K562 cells (Figure 3), we investigated the changes in MMP following stimulation with this compound. MMP disruption occurred more rapidly in Ima-R cells compared with their Ima-S counterparts. Complete membrane depolarization was achieved 10 min after DIF-3 addition in both cell lines (Figure 5A). To investigate whether MMP was connected to a loss of ATP in DIF-3-treated cells, we next determined the intracellular levels of ATP in K562 cells treated with various inhibitors of mitochondrial homeostasis. Oligomycin D, an F1/F0 ATP synthetase inhibitor, decreased the intracellular level of ATP by 25% at 6 h. An equivalent inhibition was detected in response to 20 μM DIF-3 (Figure 5B). The non-metabolizable glucose analogue 2-deoxy-D-glucose (2-DG) administered on its own reduced the intracellular ATP level by 33% at the same time, demonstrating that ATP was generated by glycolysis and not by oxidative phosphorylation in K562 cells. When used in combination, DIF-3 exerted an additive effect with 2-DG but not with oligomycin D, suggesting that DIF-3 and oligomycin D target the same pathway. Finally, the three compounds administered together exerted additive inhibitory effects on intracellular ATP levels. Importantly, we found that DIF-3 failed to inhibit the glycolytic flux over the time period tested (Figure 5C). Taking these new observations into account, we quantified the reactive oxygen species (ROS) generated by measuring superoxide production in the mitochondria (Figure 5D and 5E) and hydrogen peroxide production in the cytoplasm (Figure 5F and 5G). DIF-3 induced a rapid and robust production of ROS into the mitochondria generating ten-fold more superoxide species at 30 min that in untreated cells. At the same time, a three-fold increase in hydrogen peroxide production was detected in DIF-3 treated cells (Figure 5E and 5G). Finally, inhibition of the basal and DIF-3-mediated mitochondrial membrane potential by 10 mM TIRON, a cell permeable ROS scavenger, failed to strongly affect DIF-3-mediated changes in the mitochondrial membrane potential, suggesting that the loss of MMP precedes mitochondrial ROS production (Supplementary Figure S7).

### DIF-3 triggers caspase-independent cell death involving DRP1

**Figure 6:**
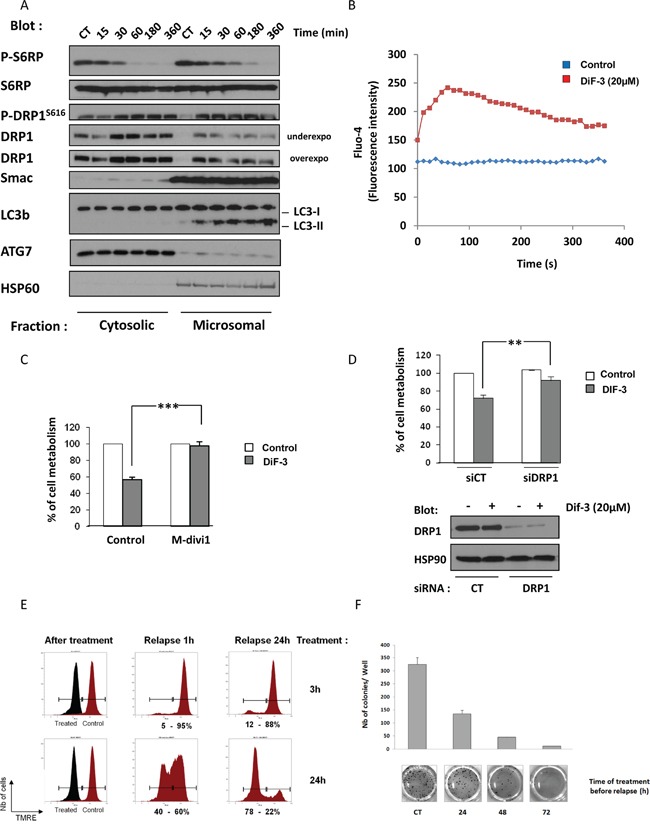
DIF-3 triggers DRP1-dependent cell death **A.** K562 cells were incubated with 20 μM DIF-3. At the times indicated, cells were harvested and washed, and subcellular fractions were prepared. Protein samples were separated via electrophoresis, and the expression of p-S6RP, S6RP, p-DRP1, DRP1, Smac and LC3-b were visualized via western blotting. As expected, HSP60 was found only in the microsomal fraction. **B.** K562 cells were loaded using the Fluo-4 Direct Calcium Assay kit and stimulated with 20 μM DIF-3. Calcium flux values are expressed as the median fluorescence intensity (MFI, in arbitrary units) and represent the mean of 3 independent experiments performed in duplicate. **C.** K562 cells were incubated with 20 μM DIF-3 alone or were preincubated with 10 μM M-divi1 and then treated with DIF-3. After 48 h, cellular metabolism was measured by using the XTT assay, as described in the Materials and Methods section. **D.** K562 cells were transfected with control or DRP1 siRNA and, 48 h later, were treated with 20 μM DIF-3 for another 48 h. Finally, cellular metabolism was measured by using the XTT assay. **E.** K562 cells were incubated with 20 μM DIF-3 for 3 or 24 h. Cells incubated with DIF-3 and untreated cells were washed, plated in fresh medium for 1 or 24 h, loaded with TMRE and then analyzed with a cytometer. The results are expressed in arbitrary units as the median fluorescence intensity. **F.** K562 cells were or were not incubated with 20 μM DIF-3 for 24, 48 or 72 h. Then, cells were washed and 10^3^ cells were placed in a methylcellulose matrix. After 10 days, the colonies were stained with MTT, counted and imaged (lower panel). Histograms represent the means ± SEM of colonies formed after the indicated treatment time (upper panel).

As mentioned previously, DIF-3 triggered a rapid loss of MMP in K562 cells (Figure 5). Unexpectedly, this decrease in MMP was not associated with caspase activation (Figure 1E). Accordingly, neither Smac (Figure 6A) nor cytochrome c redistribution (Supplementary Figure S8) from the microsomal to the cytoplasmic fraction was detected in DIF-3-treated K562 CML cells. As shown by the subcellular fractionation experiment presented in Figure 6A, DIF-3 induced the phosphorylation of DRP1 and its redistribution from the cytosolic to the microsomal fraction in as little as 15 min (Figure 6A), an event known to be associated with increased mitochondrial fission, which was already suggested by the electron microscopy images (Figure 3). In addition, the time course of DRP1 phosphorylation and redistribution into the microsomal fraction was shown to parallel that of the accumulation of LC3-II. Finally, we confirmed the rapid dephosphorylation of ribosomal protein S6, thus indicating the inhibition of the mTOR pathway (Figure 4A) in this experiment. It has previously been demonstrated that the relocation of DRP1 and its recruitment to the mitochondria is a consequence of Ca^2+^ influx [16]. We therefore decided to study calcium release in K562 cells following DIF-3 treatment. As expected, we found that DIF-3 treatment induced a strong increase of calcium release into the cytoplasm, with a maximal intensity at 1 min (Figure 6B). This rapid release of Ca^2+^ preceded DRP1 relocation and thus may explain this phenomenon.

To investigate the role of DRP1 in DIF-3-mediated cell death, K562 cells were pretreated with 10 μM of M-divi1, a specific DRP1 inhibitor, or were left untreated [35]. As illustrated in (Figure 6C), M-divi1 conferred full protection against the DIF-3-induced decrease in cellular metabolism. Moreover, knock-out of DRP1 with a specific siRNA (Figure 6D, lower panel) mimicked the effect of the pharmacological inhibitor (Figure 6D, upper panel), supporting the involvement of DRP1 in mediating the effect of DIF-3.

To determine whether the antiproliferative effect of DIF-3 was reversible, K562 cells were treated for 3 or 24 h with a maximally effective dose of DIF-3 (20 μM). They were then washed, and the reversibility of the DIF-3 effect on MMP was analyzed. All cells exhibited a complete abolition of MMP after 3 or 24 h of treatment (Figure 6E, left panel). Cells were then washed and grown in culture medium. After a 1-h relapse, cells treated with DIF-3 for 3 h exhibited a complete restoration of MMP. Conversely, only 60% of the cells treated with DIF-3 for 24 h were found to have restored their MMP (Figure 6E, middle panel). After a 24-h relapse, 90% of the cells treated with DIF-3 for 3 h and 20% of the cells treated with DIF-3 for 24 h exhibited normal MMPs (Figure 6E, right panel). This experiment shows that the effect of DIF-3 on mitochondrial function is fully reversible during the first 3 h of DIF-3 treatment. After a longer treatment period, the effects of DIF-3 became irreversible and led to growth inhibition and finally cell death.

Finally, we analyzed the potential of K562 cells to form colonies after increasing DIF-3 treatment durations (24 h, 48 h and 72 h) (Figure 6F). Cells were stimulated for the indicated times, washed and grown in methylcellulose medium for 7 days. The coloration of colonies indicated that treatment with DIF-3 for 24 h, 48 h and 72 h strikingly reduced the clonogenic potential of K562 cells by 60%, 85% and 95%, respectively.

### DIF-3 inhibits the growth of imatinib-resistant K562 cells in xenografted mice

**Figure 7:**
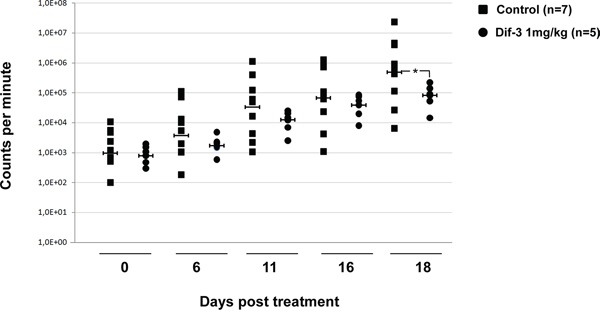
DIF-3 inhibits tumor growth in nude mice Cells (5 × 10^6^) from ImaR-K562 leukemic cell lines were implanted in both flanks of nude mice. After tumors were established, animals received a daily intraperitoneal injection of either DIF-3 (1 mg/kg body weight in PBS) or the PBS vehicle as a control. The results are expressed as the increase in tumor size as a function of time. The number of tumors found was 14 and 10 for mice treated with the control medium and DIF-3-containing medium, respectively.

Tumor cells frequently harbor defects in apoptosis and are thus less sensitive to apoptosis-inducing agents. In this context, the mode of cell death induced by DIF-3 could be used to circumvent resistance to apoptosis in cancer cells. To validate the effect of DIF-3 *in vivo*, we implanted human Ima-R K562 CML cells in the flanks of athymic mice. Mice were separated in two groups, one receiving a vehicle and the other receiving 1 mg/kg of DIF-3 five days a week. DIF-3 treatment was found to reduce the level of photon emission from tumors by approximately ten-fold relative to that of untreated tumors. Although the effect of DIF-3 was observed on all treatment days, it was clearly significant at day 18 (Figure 7). At this time point, the inhibition of tumor growth was close to 90%.

## DISCUSSION

In this study, we show that the potent anti-leukemic effect of differentiation-inducing factor 3 (DIF-3) observed *in vitro* and *in vivo* involves impaired mitochondrial function. Indeed, DIF-3 triggered a rapid rise in intracellular Ca^2+^ followed by loss of MMP and an increase in mitochondrial and cytoplasmic reactive oxygen species (ROS) production in K562 cells, without any evidence of cytochrome c or Smac release or caspase activation. The DIF-3-induced calcium release was correlated with an increase in the phosphorylation of DRP1 on threonine 616 and its translocation from the cytosolic to the microsomal fraction. Consequently, DIF-3 promoted mitochondrial fission, as assessed by confocal microscopy. All of these events culminated in caspase-independent cell death, as demonstrated by the lack of protection by the pan-caspase inhibitor zVAD-fmk from a DIF-3-mediated decrease in cellular metabolism and colony formation. Interestingly, phosphorylation of DRP1 on Ser616 has previously been reported to be a key mechanism regulating mitochondrial fission [7, 22, 35–37].

Accordingly, we show here that the pharmacological inhibition of DRP1 by M-divi1 or the depletion of DRP1 by specific siRNA dampened DIF-3′s effect on cellular metabolism, confirming the importance of DRP1 and mitochondrial fission in the effect of DIF-3. Previous studied have shown a direct interaction between DRP1 and AMPK, and AMPK silencing has also been shown to significantly reduce DRP1 phosphorylation and mitochondrial fission [38]. In this context, it would be interesting in future experiments to investigate whether DIF-3 is able to increase the interaction between DRP1 and AMPK.

Of note, it has recently been shown, using DIF-3 modified fluorescent probes, that mitochondria are the target organelles of the DIF-3 effect and that DIF-3 derivatives disturb mitochondrial activity and promote mitochondrial O_2_ consumption [39]. These findings strongly suggest that DIF-like molecules suppress cell growth and induce cell death, at least in part, by acting as mitochondrial uncouplers. In the present study, we confirmed and extended these findings by demonstrating that DIF-3 is able to induce MMP dissipation within minutes of stimulation, suggesting a direct effect of DIF-3 on mitochondria. We also demonstrated that the alkyl chain on position 1 of DIF-3 is essential for its anti-leukemic effect and therefore may interact with the mitochondrial membrane.

Another study performed on hematopoietic cells showed that following CD47-triggering, B-cell chronic lymphocytic leukemia (B-CLL) cells are efficiently killed by a caspase-independent process called necrotic cell death or type III cell death [40]. Although CD47-mediated cell death was also accompanied by the translocation of DRP1 from the cytosol to the mitochondria, as is the case for DIF-3-mediated cell death, we do not know at present whether the cell death induced by both agents is similar. The prerequisite for the induction of type III cell death in mammalian cells is not known, and the nature and exact mechanisms involved are also unclear. Recent studies by Barbier *et al.* aimed at deciphering the hallmarks of type III cell death have led to the conclusions that targeting type III cell death may be an interesting option in treating cells that exhibit defects in type I cell death (apoptosis).

One of the most striking effects of DIF-3 was a rapid induction of LC3-II conversion, which is indicative of an increase in autophagy. However, the inhibition of ATG proteins or the pharmacological inhibition of autophagy using 3-methyl adenine, bafilomycin A1 or chloroquine, failed to modulate the effect of DIF-3 on cell death, suggesting that autophagy is not directly involved in the anti-leukemic effect of DIF-3. Importantly, carbonyl cyanide m-chlorophenylhydrazone (CCCP), a mitochondrial uncoupler, has recently been reported to mediate parkin-dependent mitophagy [41, 42]. Moreover, another recent study has established that CCCP promotes mitophagy via ULK1 phosphorylation and activation [43]. Here, we established that DIF-3 failed to induce ULK1 phosphorylation at Ser555 and activation (Supplementary Figure S9). Thus, upon DIF-3 stimulation, autophagy might occur to restore cellular ATP production or as a mechanism to eliminate swollen mitochondria, independently of the regulation of cell death.

In this study we also aimed at investigating the early signaling events associated with the DIF-3 effect. One of the more striking effects of DIF-3 was the observation that this compound triggered rapid dephosphorylation and inhibition of the mTOR pathway, leading to a complete inhibition of P70S6K and S6 protein phosphorylation. It has previously been reported that ROS production leads to mTOR pathway dephosphorylation [44] in a neuronal model, suggesting that the increase of ROS production induced by DIF-3 treatment may be responsible for the effect of DIF-3 in a CML model as well. Inhibition of mTOR could thus explain the pro-autophagic effect of DIF-3 in imatinib-sensitive and resistant K562 cells. The unique ability of DIF-3 to inhibit the mTOR pathway and to induce non-apoptotic cell death is potentially of great interest in cancer therapy because cancer cells often display defects in apoptosis, and targeting other modes of cell death could contribute to eradicating tumor cells.

Finally, the observation that DIF-3 was similarly effective in Ima-S and Ima-R K562 cell lines is of great interest because targeting non-apoptotic cell death may be an interesting option in treating tumor cells that have an apoptotic defect, as has recently been suggested [40]. Another important finding of our study is the fact that DIF-3 exerted potent anti-leukemic effects *in vivo* in a mouse model of Ima-R K562 cell xenotransplantation. DIF derivatives have previously been reported to exhibit potent anti-tumoral effects *in vitro* and *in vivo* in different types of cancer [24, 28, 45, 46]. Here, we show that DIF-3 efficiently eliminates Ima-R K562 cells *in vitro* and that it exhibits potent anti-leukemic effects *in vivo*. Potentially of greater interest, we also established that DIF-3 preferentially eradicates CD34^+^ CML cells relative to its effect on CD34^−^ cells, indicating a better selectivity for leukemic than normal myeloid precursors. These results confirm that mitochondrial targeting may constitute an effective strategy for eradicating leukemia stem cells [5] [47]. To the best of our knowledge, DIF-3 or its derivatives have not been evaluated in clinical studies, but its mechanism of action and greater specificity toward leukemic cells, at least in CML cells, warrants further investigation for it use in treating this hematopoietic malignancy.

## MATERIALS AND METHODS

### Cell cultures

The human CML K562, cell line was provided by ATCC and was grown in RPMI 1630 medium (Lonza, Walkersville, MD, USA) in the presence of 5% FCS. Human HeLa cervical carcinoma cell line was grown in DMEM (Lonza) in the presence of 10% FCS. All cell cultures were grown at 37°C under 5% CO2, 50 units/ml penicillin and 50 mg/ml streptomycin to minimize contamination.

### Isolation of bone marrow patient primary

Blood samples were collected from patients newly diagnosed for CML as part of an institutionally approved cellular sample collection protocol. Informed consent has been obtained according to institutional guidelines. Mononuclear cells were isolated from blood samples by density centrifugation (Ficoll-PaqueTM Plus), washed with PBS, 5% SVF, 2 mM EDTA and resuspended in cell culture medium (IMDM, 10% fetal bovine serum) and incubated overnight at 37°C in a 5% CO2 incubator before CD34+ cells isolation. CML cells were labelled with CD34 microbeads isolated by magnetic positive selection (StemSepTM Human CD34 Selection Kit; StemCell, Vancouver, BC, Canada). Purity was estimated to At least 90% by FACS analysis. Experiments were performed using a StemSpanR SFEM medium (StemCell, Vancouver, BC, Canada) supplemented with 100 ng/ml human recombinant SCF, FLT3-L and 20 ng/ml human recombinant IL-3, IL-6 and G-CSF (Peprotech, Rocky Hill, NJ, USA).

### Reagents

Sodium fluoride and orthovanadate, phenylmethylsulfonyl fluoride, aprotinin and leupeptin were purchased from Sigma (Saint-Louis, MO, USA). Anti-phospho-mTor, anti-mTor, anti-phospho-P70S6kinase (thr389), anti-P70S6kinase, anti-phospho-S6 Ribosomal Protein (ser235-236), anti-S6 Ribosomal Protein, anti-LC3-b, anti-phospho-DRP1 (ser616) and HRP conjugated anti-rabbit antibodies were from Cell Signaling Technology (Beverly, MA, USA). Anti-Hsp60, anti-Hsp90 and anti-DRP1 antibodies were purchased from Santa Cruz Biotechnology (Santa Cruz, CA, USA). Anti-Smac antibody was purchased from RnD systems (Minneapolis, MN, USA). HRP-conjugated anti-mouse and anti-goat antibodies were from Dakopatts (Glostrup, Denmark).

### Cell metabolism assay

K562 cells (20.103 cells/100ml) were incubated in a 96-well plate with indicated concentration of STS for 6 h. About 50 ml of XTT reagent (sodium 3′-[1-(phenylaminocarbonyl)-3,4-tetrazolium]-bis (4–methoxy–6–nitro) benzene sulfonic acid hydrate) (MPP) (Roche diagnostics, Indianapolis, IN, USA) was added to each well as described previously. The absorbance of the formazan product, reflecting cell viability, was measured at 490 nm. Each assay was performed in quadruplicate.

### Cell death assays

After DIF-3 stimulation, K562 cells were stained with propidium iodide (Sigma, Saint-Louis, MO, USA). Then, staining cells were analyzed by flow cytometry.

### Colony formation assays

DIF-3 was added to K562 cell lines or primary cells (103 CD34+ or CD34- cells/ml) growing in semisolid methylcellulose medium. Metho-Cult H4100 or H4236 were used for cell lines and primary CD34+ cells respectively (StemCell Technologies Inc., Vancouver, Canada). Colonies were detected after 10 days of culture by adding 1 mg/ml of 3-(4,5-dimethylthiazol-2-yl)-2,5-diphenyltetrazolium bromide (MTT) reagent and were scored by Image J quantification software (U.S. National Institutes of Health, Bethesda, MD, USA).

### Electron microscopy

K562 cell pellets were collected, fixed with 1.6% glutaraldehyde, post-fixed in 1% OsO4, dehydrated in alcohol series, and embedded in epoxy resin. Thin sections were contrasted with uranyl acetate and lead citrate. Preparations were observed either with a Philips CM12 electron microscope operating at 80 kV (FEI, Eindhoven, The Netherlands) or with a Jeol 1400 (Tokyo, Japan) mounted with CCD cameras (Morada, Olympus SIS, Germany). Samples were analysed with Jeol 1200 XII Philipps electron microscope.

### Immunofluorescence analysis

HeLa cells were treated with 20 μM DIF-3. At indicated time, cells were fixed with 3% paraformaldehyde in PBS for 10 min, washed with PBS three times and permeabilized with 0.1% Triton X-100 in PBS for 2 min. After another washing with PBS, cells were incubated with anti-HSP60 antibody diluted in PBS, 1% BSA at RT for 1 h. Then cells were washed and incubated with secondary anti-goat antibody. Finally, DAPI was used to label the nuclei and slides containing cells were mounted and analyzed under a confocal microscope (ZEISS LSM510 META, Zeiss, Oberkochen, Germany).

### Western blots

K562 cells were lysed at 4°C in lysis buffer. Lysates were centrifuged at 10 000g for 10 min at 4°C and supernatants were supplemented with concentrated SDS sample buffer. A total of 30 mg of protein were separated on 12% polyacrylamide gel and transferred onto polyvinylidene difluoride (PVDF) membrane (Immobilon-P, Millipore, Bedford, MA, USA). After blocking non-specific binding sites, the membranes were incubated with specific antibodies, washed three times and finally incubated with HRP-conjugated antibody for 1 h at room temperature. Immunoblots were revealed using the enhanced chemiluminescence detection kit (Amersham Biosciences, Uppsala, Sweden).

### Measurement of cathepsin B activity

After stimulation, cells were lysed for 30 min at 4°C in lysis buffer (0.4 M Na Phosphate PH 6, 150 mM NaCl, 4 mM EDTA, 1 mM PMSF, 10 mg/ml aprotinin and 1% Triton X-100) and lysates were cleared at 10000 g for 15 min at 4°C. Each assay was performed with 50 μg of protein prepared from control or stimulated cells. Cellular extracts were incubated with 60 μM of z-RR-AMC as substrate in the presence or absence of 1 μM Ca074-Me at 37°C. CB activity was measured by following emission at 460 nm (excitation at 390 nm). Each experiment was performed in quadruplicates and repeated at least three times. The specific cathepsin activity was expressed in arbitrary unit per mg of protein.

### MMP assay

Mitochondrial membrane potential in living cells was analyzed using TMRE (tetramethylrhodamine-ethyl ester) (Sigma, Saint-Louis, MO, USA) to label active mitochondria. Briefly, cells were incubated with TMRE at 37°C for 30 min and directly analyzed by flow cytometry. TMRE is a cell permeant, positively charged, red-orange dye that readily accumulates in active mitochondria due to their relative negative charge. Depolarized or inactive mitochondria have decreased membrane potential and fail to sequester TMRE.

### Intracellular ATP assay

Intracellular ATP level was measured using luminescence ATP detection assay (ATPlite, PerkinElmer, USA) according to manufacturer's instructions. Data were reported as arbitrary luminometric units, measured with the microplate reader Wallac Victor multilabel counter and normalized to total protein content.

Evaluation of the glycolytic rate

1.106cells were incubated in 0.5 ml of fresh medium containing 5μCi of [5-3H] glucose (10–20 Ci/mmol; PerkinElmer Life Sciences) for 1 h at 37°C. The reaction was stopped by adding an equal volume of 0.2 N HCl.3H2O was separated from [5-3H] glucose by diffusion in an airtight container for 72 h. Diffused and undiffused tritium was measured using a 1900TR liquid scintillation analyzer (Packard) and compared with controls of [5-3H] glucose only and 3H2O only to determine the rate of glycolysis.

### Cellular subfractionation

Cells were washed with wash buffer (proteoExtract Subcellular Proteome Extraction KIT, Calbiochem, La Jolla, CA, USA). Then, cells were pelleted by centrifugation (10 min at 300 g). The wash buffer was aspirated and discarded and Extraction Buffer I was added to extract cytosolic fraction. Cells were incubated for 10min at 4°C under gentle agitation, centrifugated for 10 min at 800g. The supernatant contains the cytoplasmic fraction. Extraction Buffer II was added to the pellet and cells were incubated for 30 min at 4°C under gentle agitation. Then, cells were centrifugated for 10 min at 5500xg and the supernatant containing the microsomal fraction was collected.

This crude microsomal fraction contains the plasma membrane, mitochondria, endoplasmic reticulum, Golgi apparatus and lysosomes. A total of 30 mg of cytosolic, microsomal fractions were separated on 15% polyacrylamide gel and transferred onto PVDF membrane (Chemicon, Millipore, Billerica, MA, USA). After blocking non-specific-binding sites in saturation buffer, the membranes were incubated with specific antibodies.

### Tumor regression experiments in nude mice

Female Nude NMRI Mice (Janvier, Le Genest Saint Ile, France) were randomized into two experimental groups, each containing 7 and 5 animals respectively. Animals in both groups received a 200μl injection of 5.106 ImaR-K562 leukemia cells on both flanks. When tumors reached 100 mm3, animals were injected intraperitoneally with vehicle (PBS) or DIF-3 at dose level of 1 mg/kg body weight. The growth of leukemic cells composing the tumor was visualized every 5 days in the animal after intraperitoneal injection of 30 mg/kg luciferin (Caliper Life Sciences) by bioluminescence imaging using a Photon Imager (Biospace Lab).

### Calcium flux assay

K562 cells were loaded using a Fluo-4 Direct Calcium Assay Kit according to the manufacturer's instructions (Molecular Probes, F10471). Calcium release was analyzed by flow cytometry.

### Measurements of mitochondrial ROS levels

Mitochondrial ROS levels were measured by MitosoxRed fluorescence (Molecular Probes, Eugene, USA). K562 cells were incubated in presence of 5 μM MitosoxRed for 10 min at 37°C. Relative MitosoxRed fluorescence was measured using a flow cytometer. Results were expressed in arbitrary fluorescence units.

### Measurements of intracellular ROS levels

Intracellular ROS levels were measured by DCF-DA fluorescence (Sigma, Saint-Louis, MO, USA). K562 cells were incubated in presence of 20 μM DCF-DA for 30 min at 37°C. Relative DCF-DA fluorescence was measured using a flow cytometer. Results were expressed in arbitrary fluorescence units.

### Statistical analysis

All data are presented as the mean ± SD of at least three independent determinations. P-values were determined using the Prism V5.0b software (GraphPad, La Jolla, CA, USA). Unless stated otherwise in the figure legend, comparisons of the different groups were made with the one-way ANOVA test with Bonferroni correction. P-values of 0.05 (*), 0.01 (**) and 0.001 (***) were considered statistically significant.

## SUPPLEMENTARY FIGURES



## References

[1] Mitchell P (1961). Coupling of phosphorylation to electron and hydrogen transfer by a chemi-osmotic type of mechanism. Nature.

[2] Fernie AR, Carrari F, Sweetlove LJ (2004). Respiratory metabolism: glycolysis, the TCA cycle and mitochondrial electron transport. Curr Opin Plant Biol.

[3] Reichert AS, Neupert W (2004). Mitochondriomics or what makes us breathe. Trends Genet.

[4] Galluzzi L, Vitale I, Abrams JM, Alnemri ES, Baehrecke EH, Blagosklonny MV, Dawson TM, Dawson VL, El-Deiry WS, Fulda S, Gottlieb E, Green DR, Hengartner MO, Kepp O, Knight RA, Kumar S (2012). Molecular definitions of cell death subroutines: recommendations of the Nomenclature Committee on Cell Death 2012. Cell Death Differ.

[5] Kroemer G, Galluzzi L, Brenner C (2007). Mitochondrial membrane permeabilization in cell death. Physiol Rev.

[6] Campello S, Scorrano L (2010). Mitochondrial shape changes: orchestrating cell pathophysiology. EMBO Rep.

[7] Youle RJ, van der Bliek AM (2012). Mitochondrial fission, fusion, and stress. Science.

[8] Chen H, Chan DC (2009). Mitochondrial dynamics--fusion, fission, movement, and mitophagy--in neurodegenerative diseases. Hum Mol Genet.

[9] Gogvadze V, Orrenius S, Zhivotovsky B (2008). Mitochondria in cancer cells: what is so special about them?. Trends Cell Biol.

[10] Grandemange S, Herzig S, Martinou JC (2009). Mitochondrial dynamics and cancer. Semin Cancer Biol.

[11] Ferguson SM, De Camilli P (2012). Dynamin, a membrane-remodelling GTPase. Nat Rev Mol Cell Biol.

[12] Chan DC (2006). Mitochondria: dynamic organelles in disease, aging, and development. Cell.

[13] Wang Z, Jiang H, Chen S, Du F, Wang X (2012). The mitochondrial phosphatase PGAM5 functions at the convergence point of multiple necrotic death pathways. Cell.

[14] Jahani-Asl A, Slack RS (2007). The phosphorylation state of Drp1 determines cell fate. EMBO Rep.

[15] Chang CR, Blackstone C (2007). Cyclic AMP-dependent protein kinase phosphorylation of Drp1 regulates its GTPase activity and mitochondrial morphology. J Biol Chem.

[16] Cereghetti GM, Stangherlin A, Martins de Brito O, Chang CR, Blackstone C, Bernardi P, Scorrano L (2008). Dephosphorylation by calcineurin regulates translocation of Drp1 to mitochondria. Proc Natl Acad Sci U S A.

[17] Taguchi N, Ishihara N, Jofuku A, Oka T, Mihara K (2007). Mitotic phosphorylation of dynamin-related GTPase Drp1 participates in mitochondrial fission. J Biol Chem.

[18] Estaquier J, Arnoult D (2007). Inhibiting Drp1-mediated mitochondrial fission selectively prevents the release of cytochrome c during apoptosis. Cell Death Differ.

[19] Frank S, Gaume B, Bergmann-Leitner ES, Leitner WW, Robert EG, Catez F, Smith CL, Youle RJ (2001). The role of dynamin-related protein 1, a mediator of mitochondrial fission, in apoptosis. Dev Cell.

[20] Barsoum MJ, Yuan H, Gerencser AA, Liot G, Kushnareva Y, Graber S, Kovacs I, Lee WD, Waggoner J, Cui J, White AD, Bossy B, Martinou JC, Youle RJ, Lipton SA, Ellisman MH (2006). Nitric oxide-induced mitochondrial fission is regulated by dynamin-related GTPases in neurons. EMBO J.

[21] Twig G, Elorza A, Molina AJ, Mohamed H, Wikstrom JD, Walzer G, Stiles L, Haigh SE, Katz S, Las G, Alroy J, Wu M, Py BF, Yuan J, Deeney JT, Corkey BE (2008). Fission and selective fusion govern mitochondrial segregation and elimination by autophagy. EMBO J.

[22] Kashatus JA, Nascimento A, Myers LJ, Sher A, Byrne FL, Hoehn KL, Counter CM, Kashatus DF (2015). Erk2 phosphorylation of Drp1 promotes mitochondrial fission and MAPK-driven tumor growth. Mol Cell.

[23] Kwong L, Xie YJ, Daniel J, Robbins SM, Weeks G (1990). A Dictyostelium morphogen that is essential for stalk cell formation is generated by a subpopulation of prestalk cells. Development.

[24] Kubohara Y (1999). Effects of differentiation-inducing factors of Dictyostelium discoideum on human leukemia K562 cells: DIF-3 is the most potent anti-leukemic agent. Eur J Pharmacol.

[25] Kubohara Y (1997). DIF-1, putative morphogen of D. discoideum, suppresses cell growth and promotes retinoic acid-induced cell differentiation in HL-60. Biochem Biophys Res Commun.

[26] Kubohara Y, Hosaka K (1999). The putative morphogen, DIF-1, of Dictyostelium discoideum activates Akt/PKB in human leukemia K562 cells. Biochem Biophys Res Commun.

[27] Akaishi E, Narita T, Kawai S, Miwa Y, Sasaguri T, Hosaka K, Kubohara Y (2004). Differentiation-inducing factor-1-induced growth arrest of K562 leukemia cells involves the reduction of ERK1/2 activity. Eur J Pharmacol.

[28] Takahashi-Yanaga F, Yoshihara T, Jingushi K, Igawa K, Tomooka K, Watanabe Y, Morimoto S, Nakatsu Y, Tsuzuki T, Nakabeppu Y, Sasaguri T (2014). DIF-1 inhibits tumor growth *in vivo* reducing phosphorylation of GSK-3beta and expressions of cyclin D1 and TCF7L2 in cancer model mice. Biochem Pharmacol.

[29] Jacquel A, Herrant M, Defamie V, Belhacene N, Colosetti P, Marchetti S, Legros L, Deckert M, Mari B, Cassuto JP, Hofman P, Auberger P (2006). A survey of the signaling pathways involved in megakaryocytic differentiation of the human K562 leukemia cell line by molecular and c-DNA array analysis. Oncogene.

[30] Jacquel A, Colosetti P, Grosso S, Belhacene N, Puissant A, Marchetti S, Breittmayer JP, Auberger P (2007). Apoptosis and erythroid differentiation triggered by Bcr-Abl inhibitors in CML cell lines are fully distinguishable processes that exhibit different sensitivity to caspase inhibition. Oncogene.

[31] Fenouille N, Puissant A, Dufies M, Robert G, Jacquel A, Ohanna M, Deckert M, Pasquet JM, Mahon FX, Cassuto JP, Raynaud S, Tartare-Deckert S, Auberger P (2010). Persistent activation of the Fyn/ERK kinase signaling axis mediates imatinib resistance in chronic myelogenous leukemia cells through upregulation of intracellular SPARC. Cancer Res.

[32] Puissant A, Dufies M, Fenouille N, Ben Sahra I, Jacquel A, Robert G, Cluzeau T, Deckert M, Tichet M, Cheli Y, Cassuto JP, Raynaud S, Legros L, Pasquet JM, Mahon FX, Luciano F (2012). Imatinib triggers mesenchymal-like conversion of CML cells associated with increased aggressiveness. J Mol Cell Biol.

[33] Jacquel A, Herrant M, Legros L, Belhacene N, Luciano F, Pages G, Hofman P, Auberger P (2003). Imatinib induces mitochondria-dependent apoptosis of the Bcr-Abl-positive K562 cell line and its differentiation toward the erythroid lineage. FASEB J.

[34] Robert G, Ben Sahra I, Puissant A, Colosetti P, Belhacene N, Gounon P, Hofman P, Bost F, Cassuto JP, Auberger P (2009). Acadesine kills chronic myelogenous leukemia (CML) cells through PKC-dependent induction of autophagic cell death. PLoS One.

[35] Tanaka A, Youle RJ (2008). A chemical inhibitor of DRP1 uncouples mitochondrial fission and apoptosis. Mol Cell.

[36] Suen DF, Norris KL, Youle RJ (2008). Mitochondrial dynamics and apoptosis. Genes Dev.

[37] Yamano K, Youle RJ (2011). Coupling mitochondrial and cell division. Nat Cell Biol.

[38] Wikstrom JD, Israeli T, Bachar-Wikstrom E, Swisa A, Ariav Y, Waiss M, Kaganovich D, Dor Y, Cerasi E, Leibowitz G (2013). AMPK regulates ER morphology and function in stressed pancreatic beta-cells via phosphorylation of DRP1. Mol Endocrinol.

[39] Kubohara Y, Kikuchi H, Matsuo Y, Oshima Y, Homma Y (2013). Mitochondria are the target organelle of differentiation-inducing factor-3, an anti-tumor agent isolated from Dictyostelium discoideum [corrected]. PLoS One.

[40] Barbier S, Chatre L, Bras M, Sancho P, Roue G, Virely C, Yuste VJ, Baudet S, Rubio M, Esquerda JE, Sarfati M, Merle-Beral H, Susin SA (2009). Caspase-independent type III programmed cell death in chronic lymphocytic leukemia: the key role of the F-actin cytoskeleton. Haematologica.

[41] Narendra D, Tanaka A, Suen DF, Youle RJ (2008). Parkin is recruited selectively to impaired mitochondria and promotes their autophagy. J Cell Biol.

[42] Geisler S, Holmstrom KM, Skujat D, Fiesel FC, Rothfuss OC, Kahle PJ, Springer W (2010). PINK1/Parkin-mediated mitophagy is dependent on VDAC1 and p62/SQSTM1. Nat Cell Biol.

[43] Joo JH, Dorsey FC, Joshi A, Hennessy-Walters KM, Rose KL, McCastlain K, Zhang J, Iyengar R, Jung CH, Suen DF, Steeves MA, Yang CY, Prater SM, Kim DH, Thompson CB, Youle RJ (2011). Hsp90-Cdc37 chaperone complex regulates Ulk1- and Atg13-mediated mitophagy. Mol Cell.

[44] Chen L, Xu B, Liu L, Luo Y, Yin J, Zhou H, Chen W, Shen T, Han X, Huang S (2010). Hydrogen peroxide inhibits mTOR signaling by activation of AMPKalpha leading to apoptosis of neuronal cells. Lab Invest.

[45] Kubohara Y, Komachi M, Homma Y, Kikuchi H, Oshima Y (2015). Derivatives of Dictyostelium differentiation-inducing factors inhibit lysophosphatidic acid-stimulated migration of murine osteosarcoma LM8 cells. Biochem Biophys Res Commun.

[46] Kubokura N, Takahashi-Yanaga F, Arioka M, Yoshihara T, Igawa K, Tomooka K, Morimoto S, Nakatsu Y, Tsuzuki T, Nakabeppu Y, Matsumoto T, Kitazono T, Sasaguri T (2015). Differentiation-inducing factor-3 inhibits intestinal tumor growth *in vitro* and *in vivo*. J Pharmacol Sci.

[47] Lamb R, Ozsvari B, Lisanti CL, Tanowitz HB, Howell A, Martinez-Outschoorn UE, Sotgia F, Lisanti MP (2015). Antibiotics that target mitochondria effectively eradicate cancer stem cells, across multiple tumor types: treating cancer like an infectious disease. Oncotarget.

