# Supplementary material for: Differentiation inducing factor 3 mediates its anti-leukemic effect through ROS-dependent DRP1-mediated mitochondrial fission and induction of caspase-independent cell death
Source: Oncotarget. 2016 Mar 24;7(18):26120–36. doi: 10.18632/oncotarget.8319 (PMC5041969; doi:10.18632/oncotarget.8319)
Supplement: Supplementary file 1 [file oncotarget-07-26120-s001.pdf]

SUPPLEMENTARY FIGURES

A

Code	Chemical structure	Chemical name
#1	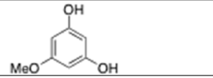	5-methoxybenzene-1,3-diol
#2	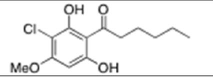	1-(3-chloro-2,6-dihydroxy-4-methoxyphenyl)hexan-1-one **(DIF-3)**
#3	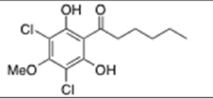	1-(3,5-dichloro-2,6-dihydroxy-4 methoxyphenyl)hexan-1-one **(DIF-1)**
#4	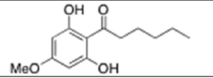	1-(2,6-dihydroxy-4-methoxyphenyl)hexan-1-one
#5	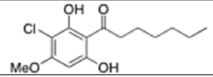	1-(3-chloro-2,6-dihydroxy-4-methoxyphenyl)heptan-1-one
#6	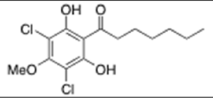	1-(3,5-dichloro-2,6-dihydroxy-4-methoxyphenyl)heptan-1-one
#7	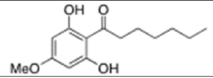	1-(2,6-dihydroxy-4-methoxyphenyl)heptan-1-one

B

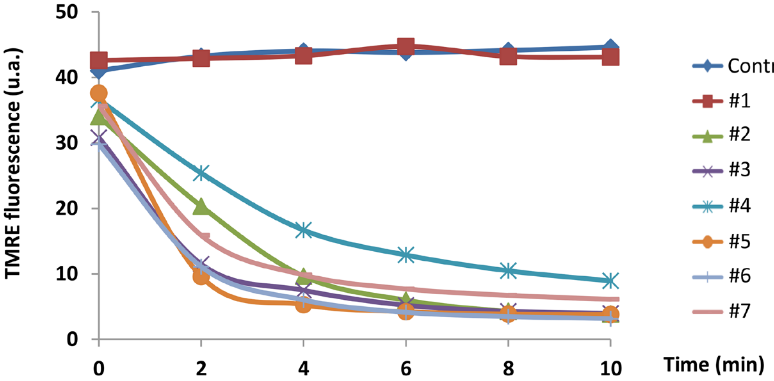

C

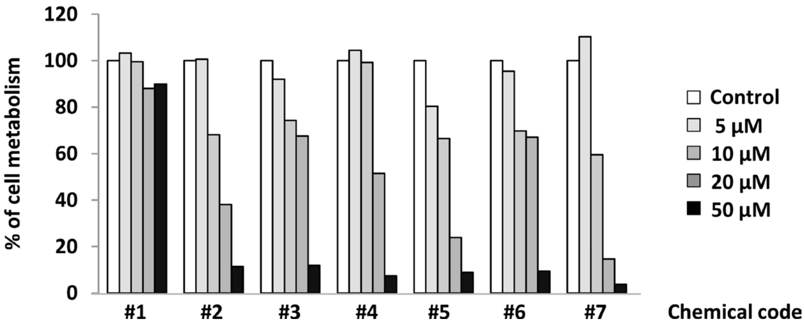

Supplementary Figure S1: DIF-3 analogs triggers a rapid decrease of Mitochondrial Membrane Potential and the consecutive alteration of cell metabolism. A. Chemical structure of the synthesized DIF analogs and chemical designation. **B.** K562 cells were loaded with TMRE for 30 min and treated with each of the seven DIF-3 analogs at a 20 µM concentration. TMRE fluorescence was evaluated by flow cytometry at 2, 4, 6, 8 and 10 min. **C.** K562 cells were stimulated with increasing concentrations of DIF-3 analogs (5, 10, 20 and 50 µM). After 24h, cell metabolism was determined using the XTT assay as described in the Material and Methods section.

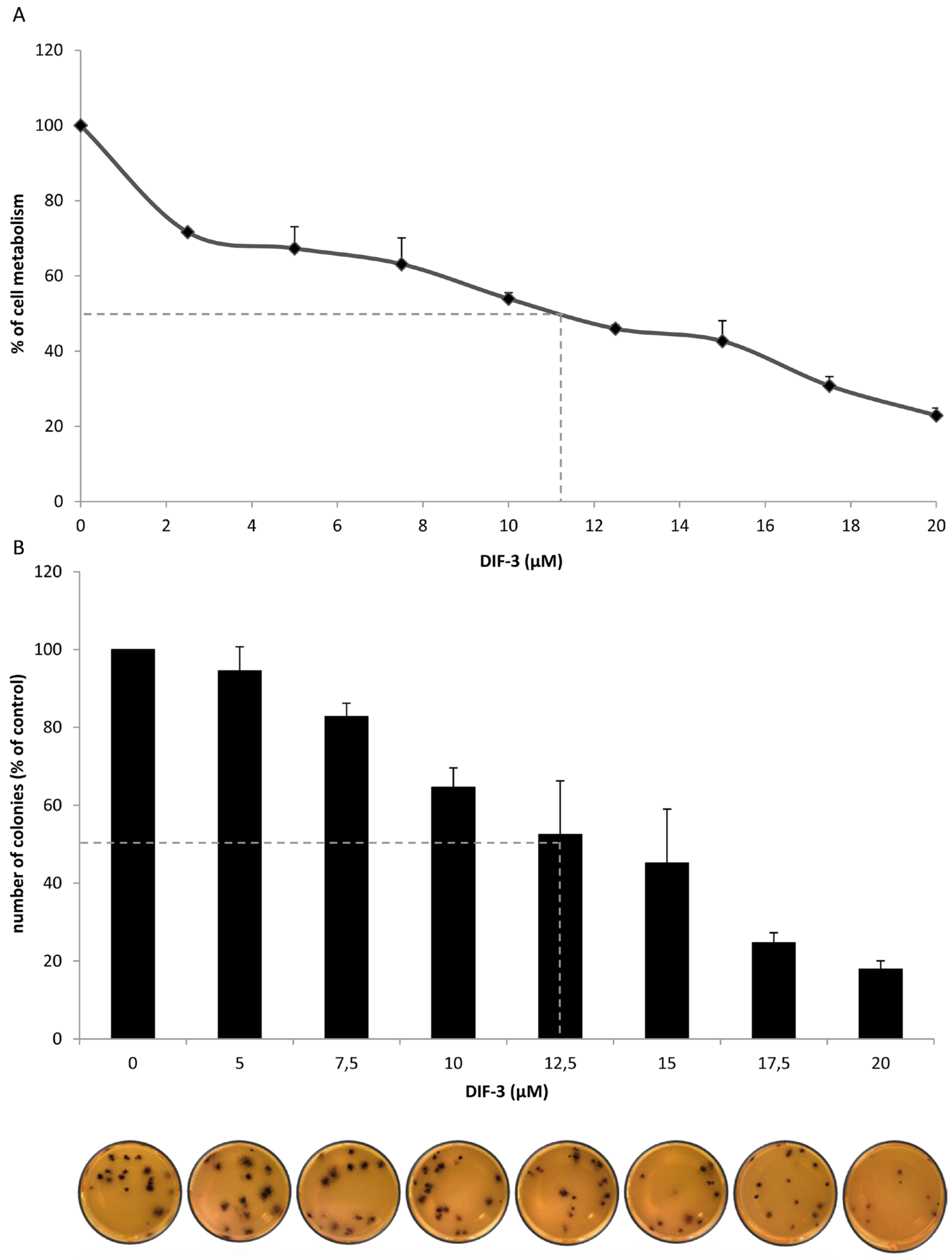

Supplementary Figure S2: DIF-3 decreases cell metabolism and the clonogenic potential of K562 cells. A. K562 cells were incubated for 48h at 37°C with increasing concentrations of DIF-3. Cell metabolism was measured by the XTT assay as described in Material and Methods section. Results are means ± SD of 3 different determinations made in triplicate. Error bars = 95% confidence intervals. **B.** Increasing concentrations of DIF-3 5 to 20µM were added to K562 CML cell lines growing in semi-solid methyl cellulose medium (0.5 x 10^3 cells/ml). Colonies were detected after 10 days of culture by adding 1 mg/ml of the MTT reagent and were scored by Image J quantification software. Results are expressed as the number of colony forming cells by well after drug treatment. Results are means ± SD of 3 different determinations made in triplicate. Error bars = 95% confidence intervals.

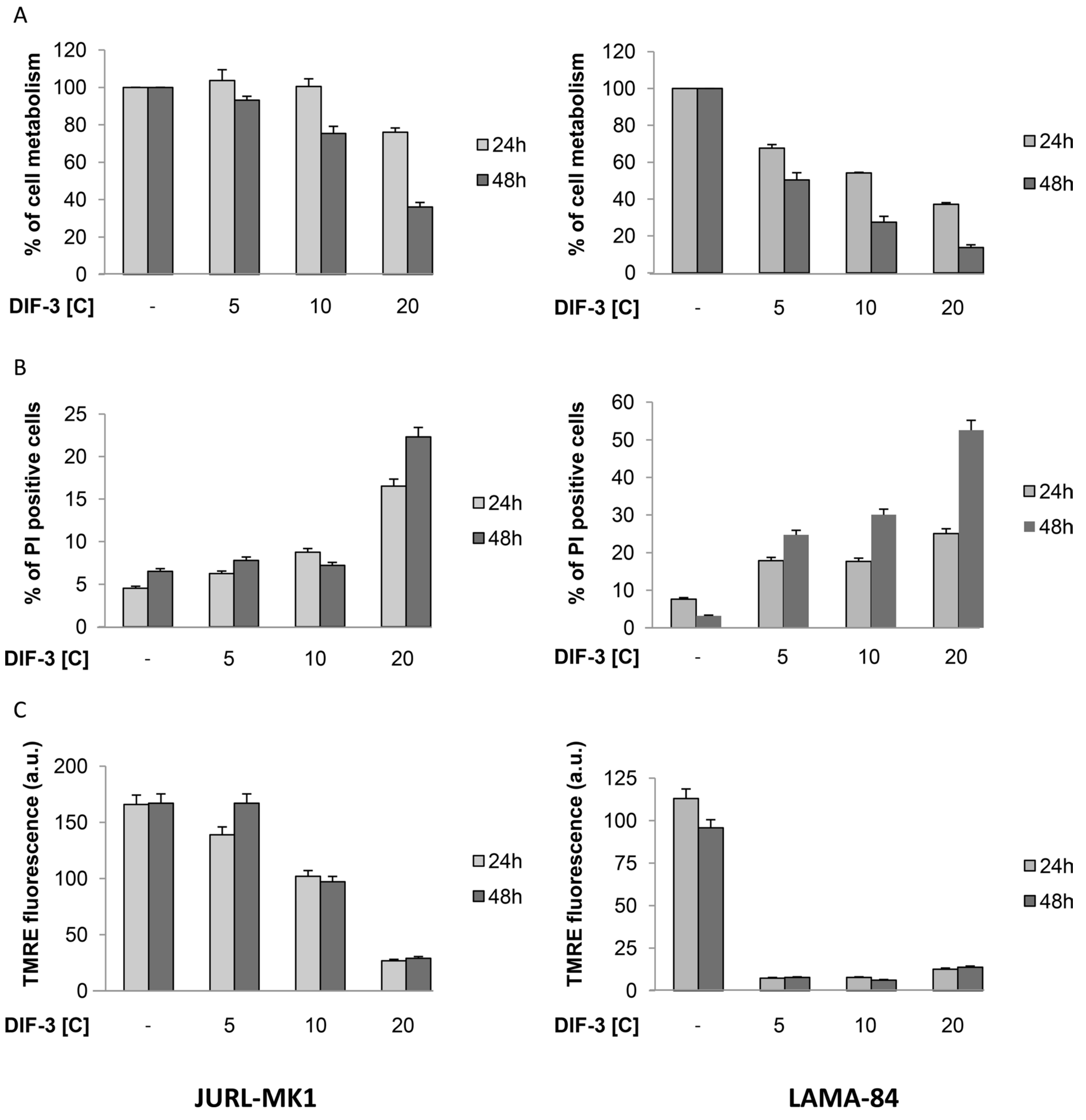

Supplementary Figure S3: DIF-3 decreases the viability of different CML cell lines. A. JURL-MK1 and LAMA-84 cells were incubated for 24 or 48 h at 37°C with increasing concentrations of DIF-3. Cell metabolism was measured by the XTT assay as described in Material and Methods section. Results are means ± SD of 3 different determinations made in triplicate. Error bars = 95% confidence intervals. **B.** JURL-MK1 and LAMA-84 cells were treated as described in Fig. 3A. After 24 and 48 h, cells were stained with Propidium Iodide and cell death analyzed by flow cytometry. **C.** JURL-MK1 and LAMA-84 cells were treated as described above. After 24 and 48 h cells were loaded with TMRE. TMRE fluorescence was analyzed using a flow cytometer. Results are expressed as arbitrary units (a.u.) and are means ± SD of 4 independent experiments performed in quadruplicate. Error bars = 95% confidence intervals.

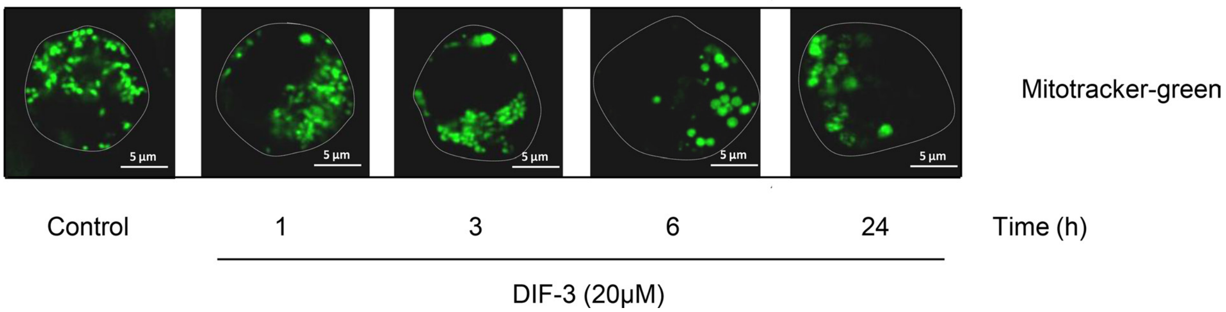

Supplementary Figure S4: DIF-3 remodel mitochondrial network in K562 cells. K562 cells were incubated at 37°C with 20 µM DIF-3. After 1, 3, 6, or 24 h cells were incubated in the presence of 200 nM of Mitotracker Green for 30 min at 37°C. Then, cells were washed and visualized by confocal microscopy.

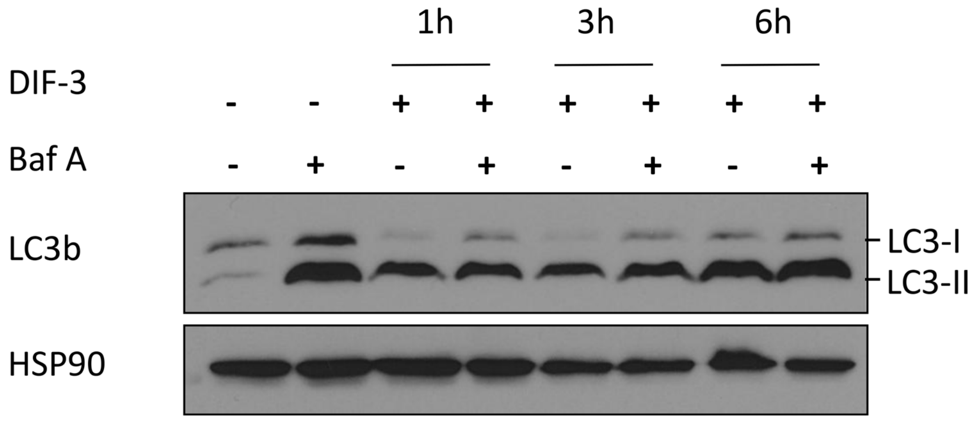

Supplementary Figure S5: DIF-3 fails to affect the autophagic flux in K562 cells. K562 cells were treated with 20 µM DIF-3 for the indicated time in either the presence or absence of Bafilomycin A1 (10 nM). Whole-cell lysates were prepared, and expression of LC3-b was visualized by western blotting. HSP90 was used as a loading control.

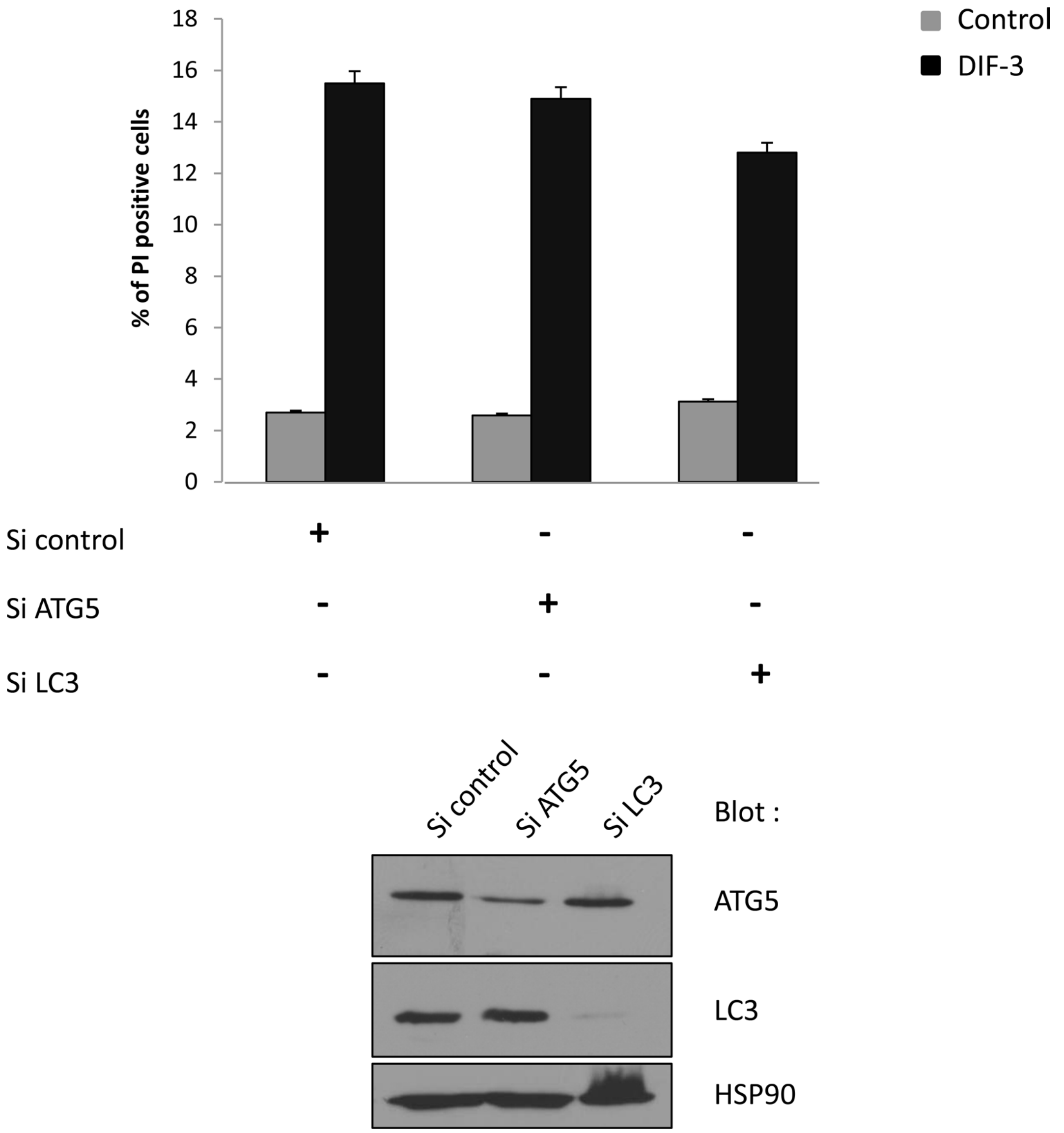

Supplementary Figure S6: Knock-down of autophagy genes fails to inhibit the DIF-3 effects on cell death. Upper panel: K562 cells were transfected with control, ATG5 or LC3 siRNA. 72 h later, cells were treated with 20 µM DIF-3 for 48h and finally stained using Propidium Iodide. PI stained cells were analyzed using a flow cytometer. Lower panel: Cells were collected 5 days after transfection. Whole-cell lysates were prepared, and the expression of ATG5 and LC3-b was visualized by western blotting. HSP90 was used as a loading control.

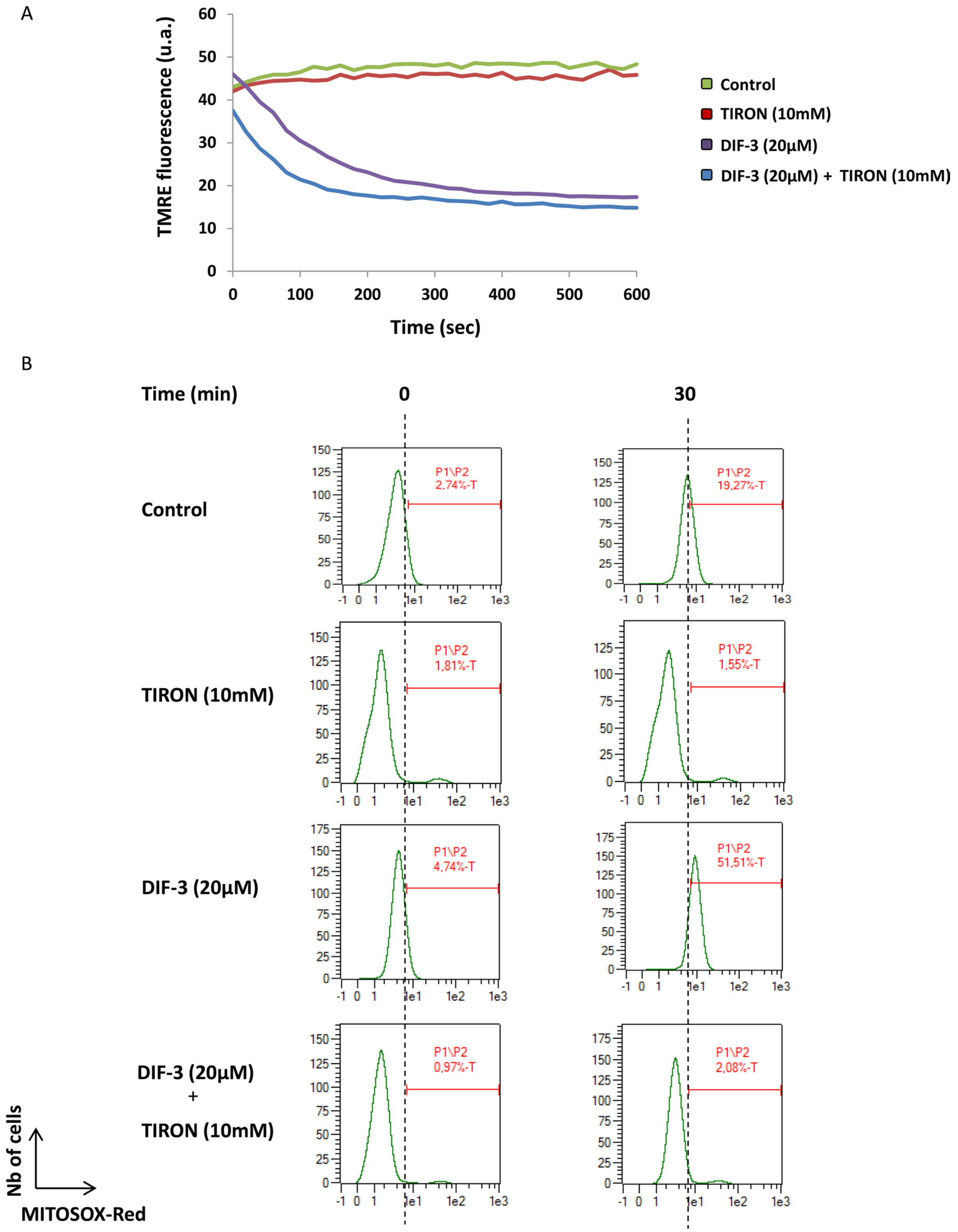

Supplementary Figure S7: MMP drop precedes ROS formation following DIF-3 treatment. A. K562 cells were loaded with TMRE and stimulated with 20 µM DIF-3 in either the presence or the absence of 10 mM TIRON. TMRE fluorescence was monitored with a flow cytometer as a function of time. **B.** K562 cells were loaded with 5 µM MITOSOX-Red during 30 min. Cells were then treated with 20 µM DIF-3 in the presence or the absence of 10mM TIRON. 30 min after stimulation, fluorescence was analyzed with a flow cytometer to quantify mitochondrial ROS formation.

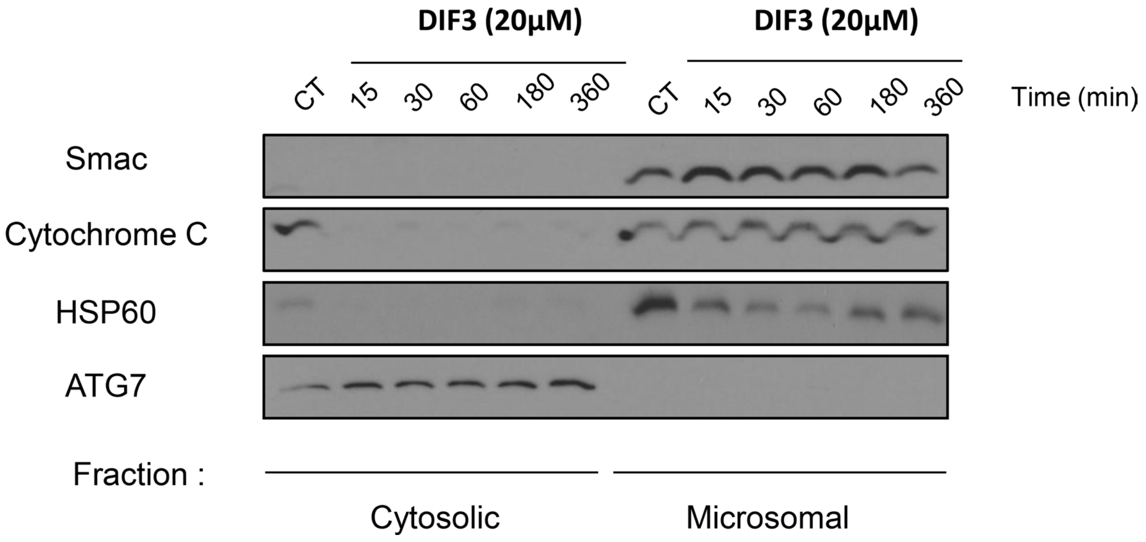

Supplementary Figure S8: DIF-3 treatment fails to induce Cytochrome c or Smac release. K562 cells were incubated with 20 µM DIF-3. At the times indicated, cells were harvested and washed, and subcellular fractions prepared. Protein samples were separated by gel electrophoresis, and the expression of cytochrome c and Smac were visualized by western blotting. As expected, ATG7 and HSP60 were found only in cytosolic and microsomal fraction, respectively.

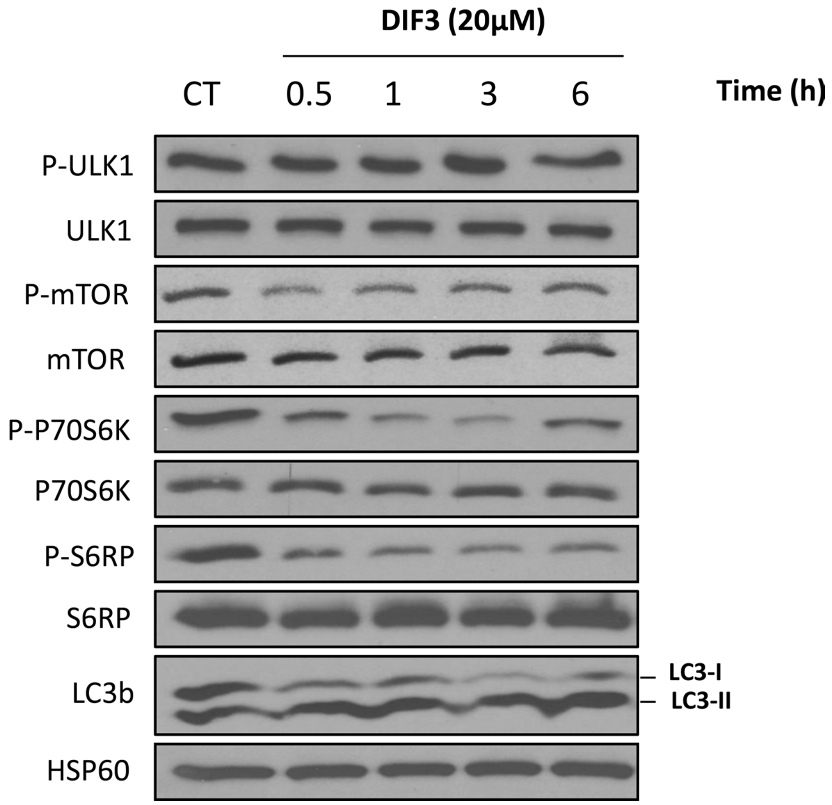

Supplementary Figure S9: DIF-3 inhibits mTOR signaling pathway. K562 cells were treated with 20 µM DIF-3 for the indicated times. Whole-cell lysates were prepared, and the expression of LC3-b, ULK1, mTOR, P70S6 kinase and S6 ribosomal protein was visualized by western blotting. The phosphorylated forms of ULK1, mTOR, P70S6 kinase and S6 ribosomal protein were also visualized by western blotting. HSP60 was used as a loading control.
